# Construction of a smart face recognition model for university libraries based on FaceNet-MMAR algorithm

**DOI:** 10.1371/journal.pone.0296656

**Published:** 2024-01-11

**Authors:** Yan Liu, Yan Qu

**Affiliations:** Library, Yantai Vocational College, Yantai, China; CCET: Chandigarh College of Engineering and Technology, INDIA

## Abstract

The continuous development of science and technology has led to the gradual digitization and intelligence of campus construction. To apply facial recognition technology to construct smart libraries in higher education, this study optimizes traditional facial recognition algorithm models. Firstly, a smart management system for university libraries is designed with facial recognition as the core, and secondly, the traditional FaceNet network is optimized. Combined with MobileNet, Attention mechanism, Receptive field module and Mish activation function, the improved multitask face recognition convolutional neural network is built and used in the construction of university smart library. The performance verification of the constructed model shows that the feature matching error value of the model in a stable state is only 0.04. The recognition accuracy in the dataset is as high as 99.05%, with a recognition error as low as 0.51%. The facial recognition model used in university smart libraries can achieve 97.6% teacher satisfaction and 96.8% student satisfaction. In summary, the facial recognition model constructed by this paper has good recognition performance and can provide effective technical support for the construction of smart libraries.

## I. Introduction

Face recognition technology is an artificial intelligence technology designed to identify individuals by analyzing face images. In face recognition technology, various sensors and algorithms are often utilized to automatically extract information from a face database to match personal identity information for face recognition purposes [1]. The main application areas of face recognition technology include identity verification, video surveillance, security systems, and healthcare, etc. Currently, the technology has been widely used in a variety of situations, such as e-commerce, smart home, network security, etc. [2]. In addition, many research institutes and companies are developing and improving face recognition technology and are constantly introducing new algorithms and hardware systems.For example, companies such as Microsoft and Google have introduced their own facial recognition systems and are constantly improving and enhancing their performance. Despite the many advances in face recognition technology, there are still some challenges, such as the impact of external factors on face recognition accuracy, such as changes in lighting, expression and posture, and recognition errors due to the poor performance of some neural networks. Therefore, the current face recognition technology needs to be further improved.

The smart library utilized in colleges and universities functions on the basis of artificial intelligence and Internet of Things technology. Its purpose is to supply teachers and students with more efficient, personalized literature and information services [3]. The smart library integrates various literary and informational resources through intelligent algorithms and data analysis. It provides intelligent and personalized literature and information services to teachers and students to enhance resource utilization and bolster their knowledge acquisition capabilities. In addition, the library utilizes digitization, automation, and intelligence technologies with features like intelligent recommendations and data mining to make it easier for readers to access required information [4, 5]. During the development of an intelligent library system at the university, implementing an intelligent access control function for the library is a significant undertaking. The incorporation of an intelligent access control management system enhances the library’s overall level of automation while also significantly reducing the need for human and material resources. In general, the utilization of face recognition technology in university libraries is widespread and can enhance security management, reader identity authentication, and service levels.

Based on this background, the study aims to optimize current face recognition technology for use in the access control identification system of the smart library. The primary objective is to enhance the accuracy and efficiency of library access control identification. Developing an intelligent face recognition model for college libraries serves two critical roles—the first being to address security concerns in libraries and the second being to improve service efficiency. The intelligent facial recognition model can offer robust security functions and assist in resolving security issues within the library. Furthermore, the developed facial recognition model can readily and accurately detect and identify individuals, ensuring solely authorized access to the premises. In comparison to conventional security measures, such as keycards or passwords, facial recognition technology proves to be more challenging to impersonate, given that each person possesses distinctive facial features. Thus, the facial recognition model enhances user experience while also substantially reducing costs associated with human resources and time.

This paperis divided into four parts. The first step involves analyzing and summarizing the existing research in the area of facial recognition-enabled smart home libraries, both nationally and internationally. The second part is to study the application of improved multi-task face recognition convolutional neural network (FaceNet-MobileNet-Mish-Attention-Receptive Field Block, FaceNet-MMAR) in the intelligent face recognition of university libraries. Firstly, the FaceNet algorithm is improved and the final face recognition model is proposed. Secondly, the intelligent management system of university library is designed. In the third part, the performance test and application analysis of the proposed model are conducted. The final part provides a summary of the experimental findings presented in this paper and suggests potential areas for future research.

## II. Related work

Computer vision is one of the hotspots. FR-tech receives widespread attention from various industries for its extensive applications in business, daily life, and government fields. Among them, the construction of intelligent facial recognition models in university libraries is a hot topic of recent research. Although the current face recognition has achieved high-precision confirmation, the efficiency of face recognition is still significantly reduced when disturbed by various conditions such as light, facial expression, posture, etc. [6]. Many scientists, researchers, and companies have proposed various improvements or innovative methods to address the challenges faced by traditional facial recognition. Wang S et al. creatively proposed a robust block diagonal dictionary grounded on virtual samples and applieditto face recognition to address the adverse effects of noise on facial recognition. After extensive comparison with many advanced facial recognition methods, it was ultimately provedthat this method greatly improves facial recognition [7]. Chen T et al. first proposed a feature fusion algorithm called centrosymmetric local binary pattern gradient direction histogram. Compared to other recent algorithms, this algorithm can efficiently and accurately recognize faces even in complex lighting conditions [8]. Paul K C and Aslan S proposed an optimized real-time facial recognition system to enhance AI facial recognition with an accuracy of 60.60% and 95% at 15 and 45 pixels, respectively [9]. This real-time system can capture low-resolution images, decreasing the possibility of unlawful activities. Bala et al. proposed a novel facial recognition algorithm to tackle ineffective recognition caused by lighting artifacts in image analysis. Experiments on the Yale B database have shown that this new algorithm significantly improves the face recognition rate even in the presence of lighting artifacts [10]. Otani Y and Ogawa H have constructed an AIFR-sys to accelerate the widespread use of AI in field research. This system’s significant advantage is the ability to incorporate additional data, which solves the problem of requiring a vast number of annotated learning graphics in the current system construction. Therefore, they consider this system to be a catalyst for accelerating the development of personal AI recognition systems [11].

Liu Y and Chen J designed and compiled a good adversarial network and multi-factor joint normalization network, while normalizing multiple complex factors, which is to convert non-frontal faces into frontal faces to improve the recognition rate. The system uses identity perception loss with convolutional neural networks (CNN) to improve face recognition performance. Complex factors are normalized to minimize interference in face recognition [12]. Ni H designed a face recognition method by refining the LeNet-5 CNN structure. This enhanced optimization yields not only short training times, but also high recognition accuracy that improves with more samples. This also proves the reliability of CNN in facial recognition and contributes to the further development of intelligent facial recognition [13]. Venugopal K R et al. designed a face recognition model utilizing discrete cosine transform, artificial neural network, and mean covariance windowing technology. The model achieves a higher recognition rate compared to traditional methods while reducing computational complexity and the number of features. This provides a foundation for the development of intelligent facial recognition [14]. Huang Y and Hu H discovered that the aging process can significantly impact facial appearance, thereby affecting the recognition rate of facial recognition. For this reason, they developed an age adversarial CNN with parallel network architecture. This system extracts particular features that are invariant to age changes through adversarial training in an age-discriminative network. The effectiveness and superiority of the age adversarial CNN was demonstrated through large-scale experiments with challenging aging face data [15].

In summary, although scholars and scientists have designed numerous systems and algorithms to improve efficiency and accuracy, few have examined the application of facial recognition algorithms in building smart university libraries. This paper will use FaceNet as a basis to optimize facial recognition accuracy and, ultimately, utilize the optimized FaceNet network to construct a smart library FR-system. For locations such as libraries with significant foot traffic, the potential application value of FR systems that are more efficient, quicker, and have superior recognition accuracy is significant.

## III. Application of FaceNet-MMAR in smart face recognition in university librarie

Traditional FaceNet networks encounter issues with large parameter quantities, complex calculations, and high model memory consumption. To achieve better precision in facial recognition for university smart library systems, the paper initially analyzed the functional modules of the library access control system and subsequently designed a library smart management system based on the analysis. On this basis, traditional FaceNet is optimized by using MobileNet as the primary feature network and Mish function as the activation function. The final FaceNet MMAR model is constructed by including the attention module and the receptive field module to enable intelligent facial recognition in university libraries.

### A. Concept of intelligent management system for university library

**Fig 1:**
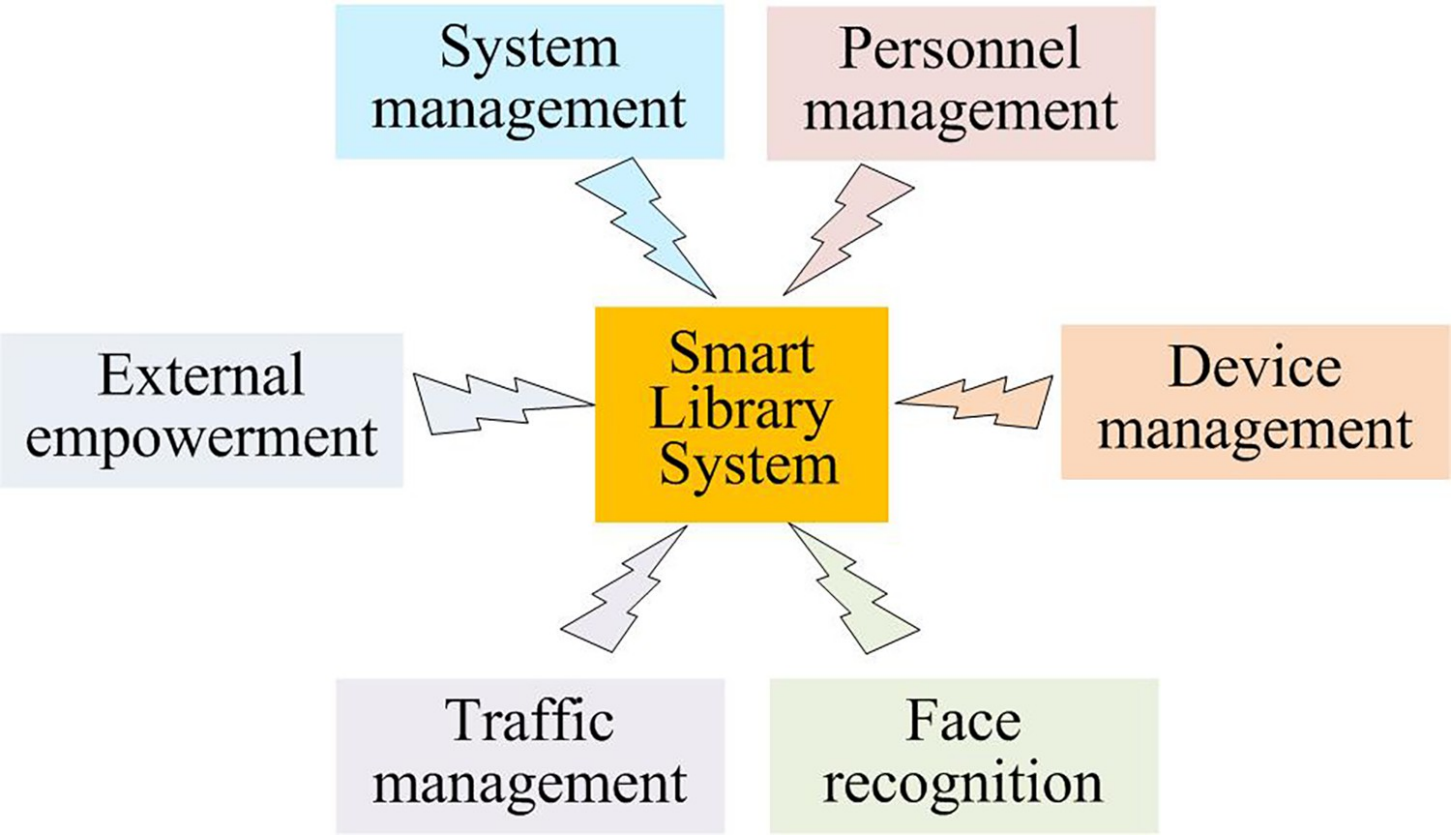
System structure of smart libraries in universities.

Currently, the management challenges facing intelligent libraries in universities are related to several content areas. First, data management lacks effectiveness. Second, space management falls short of intelligent standards. Third, resource management lacks scientific effectiveness. Fourth, user experience needs improvement. Fifth, it is necessary to improve the traffic management mode [1, 16]. To develop an intelligent management system for university libraries, the study examined the library requirements of university students and created a smart university library system featuring facial recognition technology, as illustrated in Fig 1.

**Fig 2:**
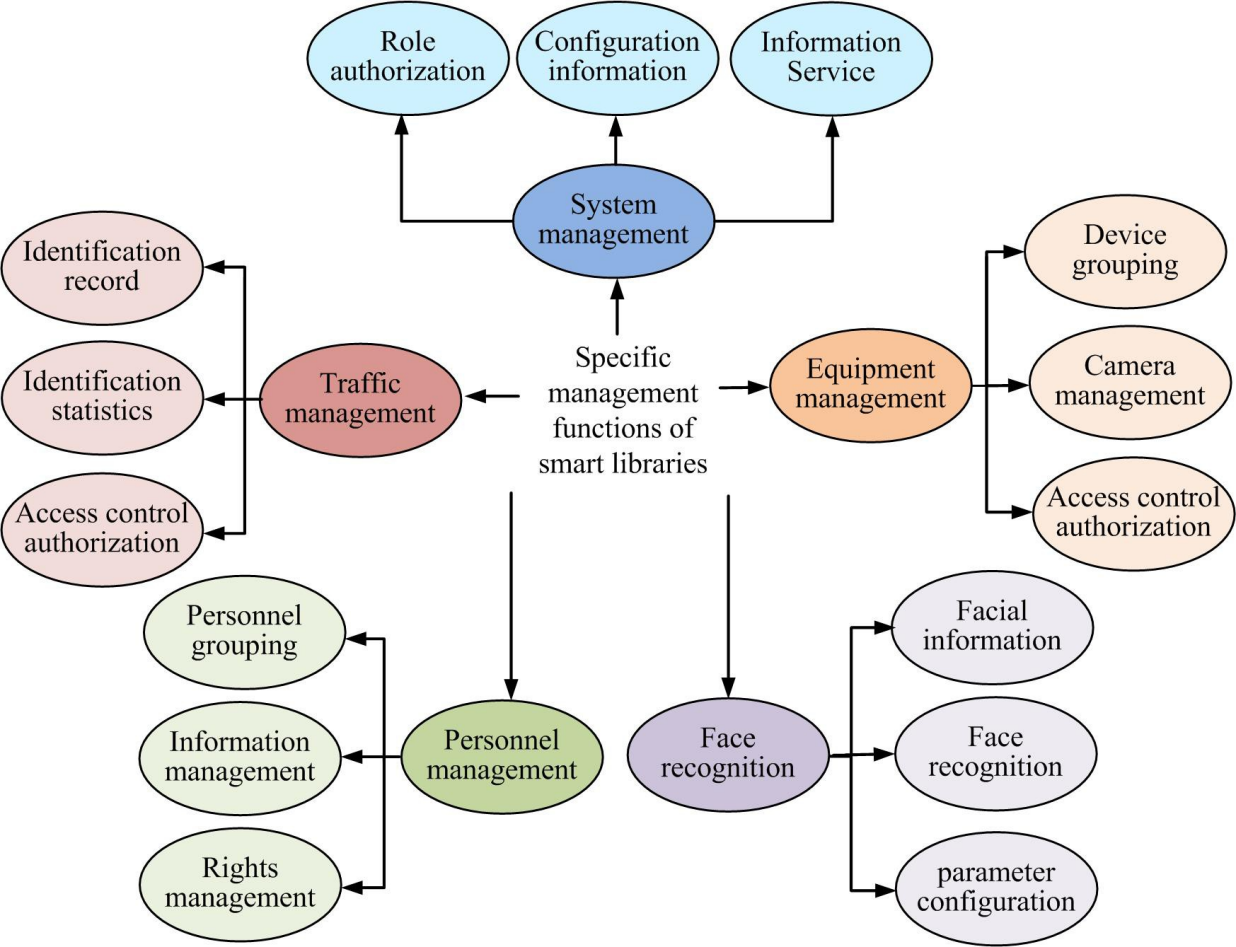
Schematic diagram of specific management functions of smart library.

From Fig 1, it is recommended that the university smart library system comprises six modules: system management, personnel management, equipment management, facial recognition, access management, and external empowerment [17]. The automatic recognition of entry and exit, as well as facial recognition for borrowing and returning books in the smart library, is achieved by establishing appropriate platforms and utilizing its implementation to support external users. Fig 2 illustrates the specific procedures of each management function.

**Fig 3:**
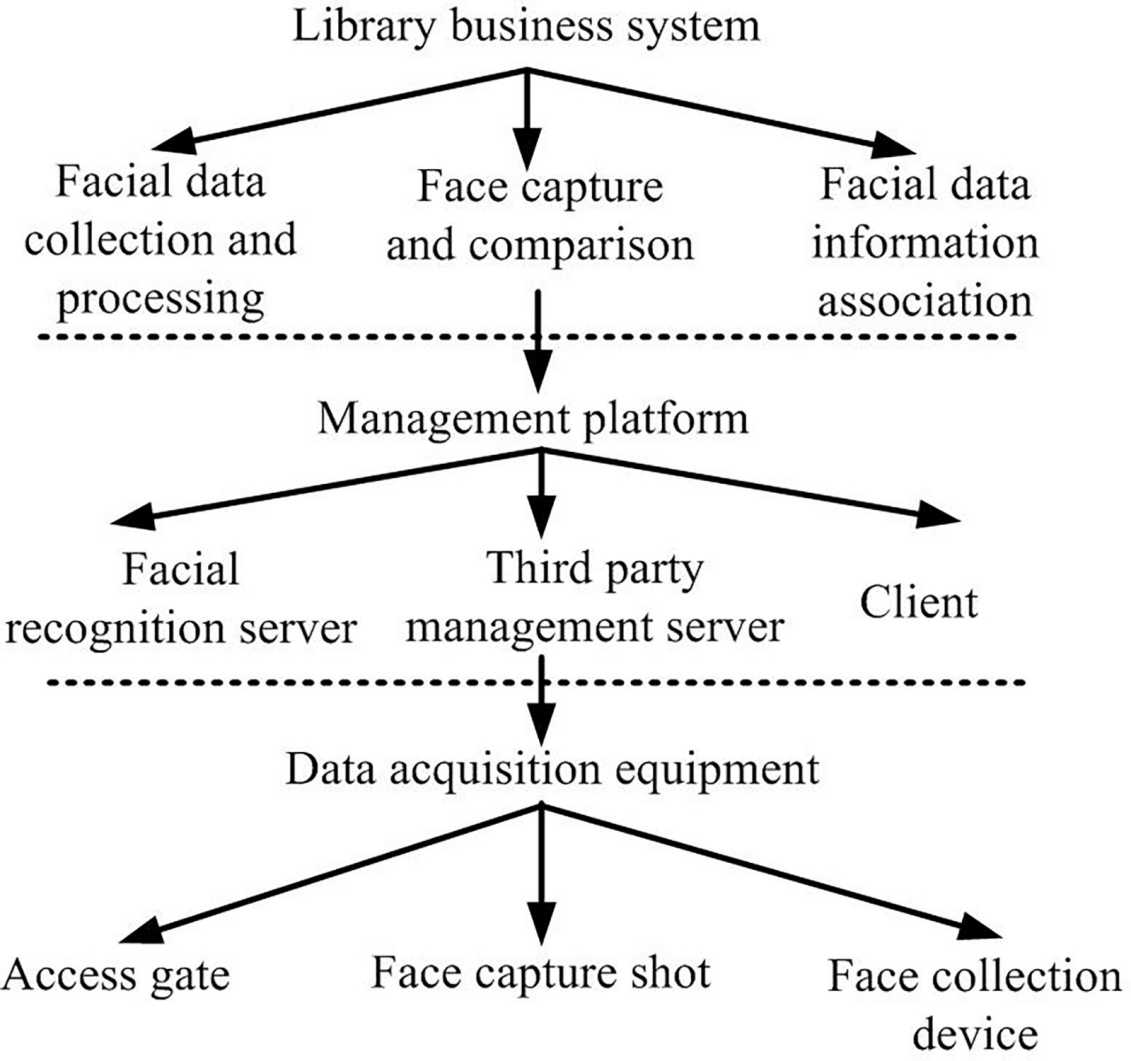
Topological map of smart library network construction.

Fig 2 presents a schematic diagram detailing the specific management functions of the smart library. Within the personnel management module, librarians must group library visitors and book borrowers, and then manage borrowing permissions along with specific book information. In the personnel management module, administrators must categorize library personnel entering and exiting, as well as personnel borrowing books. Specific book information and borrowing privileges should be managed accordingly.The device management module mainly includes device grouping, access authorization, and camera management, aiming to complete the construction of a smart library facial recognition platform by managing relevant access recognition devices. In the system management module, administrators need to perform related tasks such as role authorization, system configuration management, and log query. In the face recognition module, it is necessary to ensure that the builtface recognition platform can query face information and statistics, and perform face recognition tasks based on the set parameters. For the intelligent library system in universities, in addition to building the above functional modules, it is also necessary to build the network topology as shown in Fig 3 to ensure the normal and orderly operation of the system.

Fig 3 shows the network construction topology structure of the smart library. To build the final smart library system, the hardware construction of the entire system includes equipment acquisition, communication network, and server layer. The library business system settings include functions such as data collection, identity recognition, and facial capture. The library management platform comprises a facial recognition platform server, a third-party management platform server, and a client. The front-end data collection equipment consists of import and export face brushing gate equipment, facial capture equipment, and facial data collection equipment. By establishing the aforementioned hardware facilities and utilizing facial recognition algorithms, a comprehensive smart library management system for the university can be created. It is crucial to suggest an effective and reasonable facial recognition algorithm for this purpose.

### B. Improved FaceNet face recognition algorithm for smart library management

Local binary algorithm, eigenface recognition algorithm (ERA) and linear discriminant algorithm (LDA) are classic face recognition algorithms. With the continuous improvement of neural networks, many face recognition technology companies have begun to combine deep learning algorithms with neural networks to optimize the recognition accuracy and efficiency of traditional face recognition algorithms [8]. ERA is a biometric technology that analyzes facial images, extracts facial features, and then uses a specific matching or classification method to determine the facial identity in the image. ERA includes four steps: image preprocessing, feature matching and extraction, and recognition classification. Commonly used ERAs include ERA under deep learning and ERA under machine learning. This study optimizes on the basis of ERA to improve the recognition accuracy of existing facial recognition algorithms. In ERA, face features need to be vectorized first [18], and the expression method of face feature vector is Eq (1).


$$S=\{\Gamma_{1},\Gamma_{2},\Gamma_{3},\cdots ,\Gamma_{m}\}$$ (1)

In Eq (1), *S* represents the set of all facial images in a person’s face dataset. *m* is the facial image numbers in the dataset. Γ is the transformation of a facial image into an *N*-dimensional vector for representation. After obtaining all facial feature vectors in the dataset, calculating their average vector and recording it as Ψ.


$$\Phi_{i}=\Gamma_{i}-\Psi$$ (2)

Eq (2) is the calculation equation for the difference Φ between the vector of each facial image and the average vector. The average vector value is subtracted from the vector value of the *i*-th image to obtain the difference between the image and the average vector [19, 20].


$$\lambda_{k}=\frac{1}{M}{\sum_{n}^{m}\left(u_{k}^{}\Phi_{n}\right)}^{2}$$ (3)

Eq (3) is the distribution description equation for the average vector difference Φ. *λ*_*k*_ represents the characteristic value. *u*_*n*_ represents the unit vector corresponding to the *m* person’s face image. *u*_*k*_ represents the *k*-th vector in *u*_*n*_.


$$u_{l}^{T}u_{k}=\{\begin{array}{cc} 1 & l=k\\ 0 & l\neq k \end{array}$$ (4)

Eq (4) is the limiting condition for *u*_*k*_. $u_{l}^{T}$ represents the orthogonal form of *u*_*k*_. By using Eq (4), *u*_*k*_ can be transformed into a unit orthogonal vector, and calculating the value of *u*_*k*_ is equivalent to calculating the eigenvector value of the covariance matrix.


$$C=\frac{1}{m}\sum_{n=1}^{m}\Phi_{n}\Phi_{n}^{T}=AA^{T}$$ (5)

Eq (5) is the condition that needs to be met to calculate the eigenvectors of the covariance matrix. In Eq (5), $A=\{\Phi_{1},\Phi_{2},\Phi_{3},\cdots ,\Phi_{n}\}$. *A*^*T*^ and $\Phi_{n}^{T}$ represent the matrix forms of Φ_*n*_ and *A*, respectively. The eigenvector of the face can be obtained fromequation (1) to (5). At this time, a new face is introduced and represented with a eigenface [21]. The calculation equation is Eq (6).


$$\omega_{k}=u_{k}^{T}(\Gamma -\Psi )$$ (6)

In Eq (6), $u_{k}^{T}$ represents the matrix form of the *k*-th eigenface, and *ω*_*k*_ represents the weight of the *k*-th eigenface.


$$\Omega^{T}=[\omega_{1},\omega_{2},\cdots ,\omega_{m}]$$ (7)

In Eq (7), Ω^*T*^ represents the vector set of all eigenface weights. The calculation equation for facial recognition based on feature vectors is Eq (8).


$$\varepsilon_{k}={\left\|\Omega -\Omega_{k}\right\|}^{2}$$ (8)

In Eq (8), Ω and Ω_*k*_ represent the face to be recognized and the face of the *k*-th person in the training dataset, respectively. *ε*_*k*_ represents the Euclidean distance between two faces. When the Euclidean distance is less than the threshold, it indicates that the face to be recognized is consistent with the *k*-th person’s face in the training dataset.

**Fig 4:**
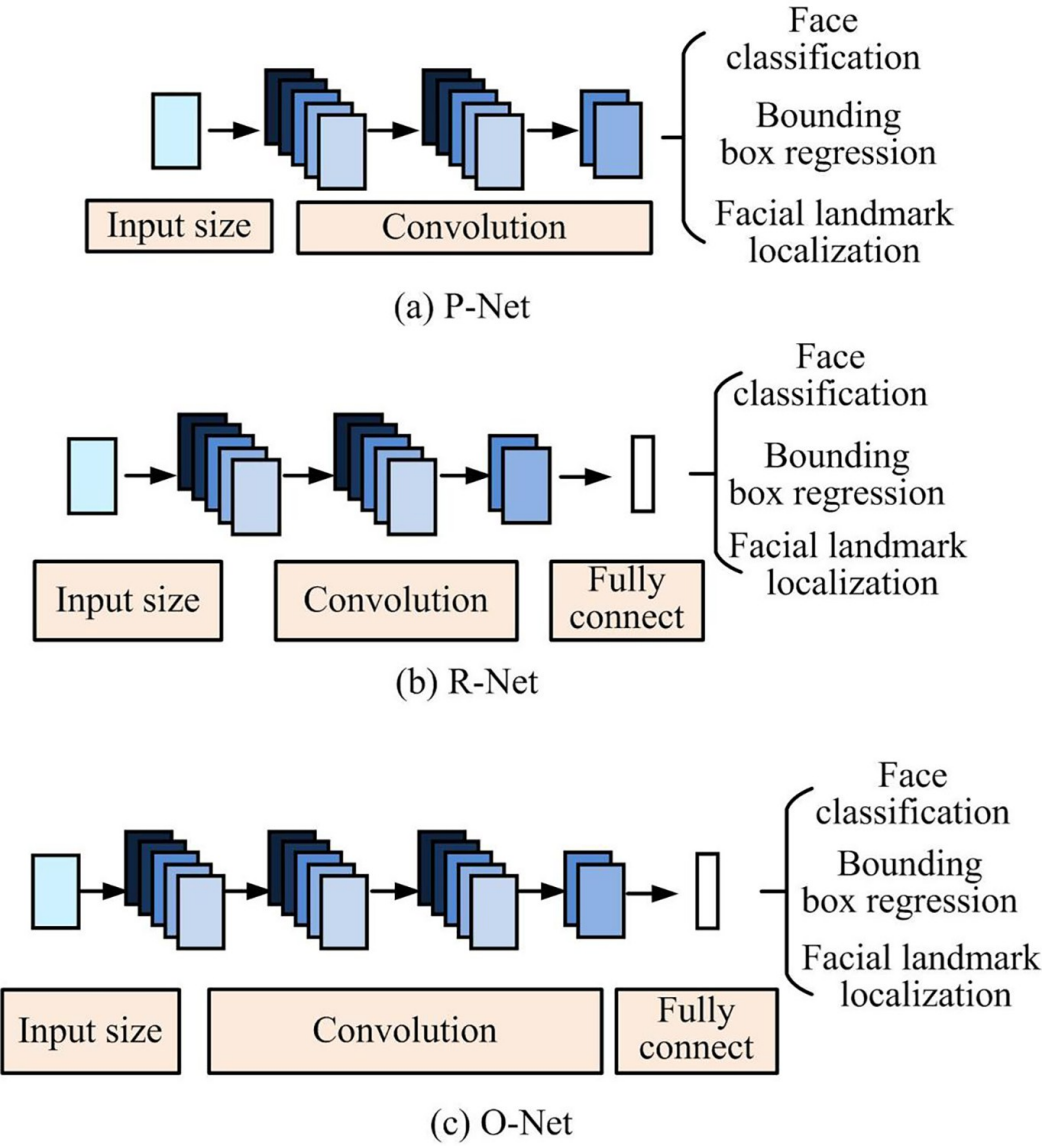
Schematic diagram of P-Net, R-Net, O-Net models.

In terms of ERA, the Multi-task CNN (MTCNN) was proposed with the aim of achieving face detection. Three sub-networks perform recognition training on the original image, with the addition of the FaceNet network to further complete face recognition. Fig 4 displays the schematic diagrams of P-Net, R-Net, and O-Net models.

Fig 4 is a schematic diagram of the P-Net, R-Net and O-Net models. P-Net is composed of a fully convolutional network that proofreads all candidate windows by utilizing boundary regression. Non-maximum suppression is employed to merge and overlap all candidate windows. R-Net is primarily utilized to improve candidate windows, and candidate windows corrected by P-Net also undergo R-Net filtering to reject inappropriate candidate windows. O-Net is mainly used to select and locate characteristic points of the final output face frame. By utilizing O-Net, the position of the feature points’ output can be determined to facilitate the succeeding facial recognition process. The face recognition equation is shown in Eq (9) of the MTCNN algorithm [22].


$$L_{i}^{\det}=-(y_{i}\log(p_{i})+(1-y_{i}^{\det})(1-\log(p_{i})))$$ (9)

In Eq (9), $L_{i}^{\det}$ represents the cross entropy loss function, and the value of this function is calculated to determine whether a face or not. $y_{i}^{\det}$ means the amount of truly recognized labels in the region. *p*_*i*_ is the probability of facial appearance. *y*_*i*_ represents the number of all labels [23].


$$L_{i}^{box}={\left\|{y^{\prime }}_{i}^{\det}-y_{i}^{\det}\right\|}_{2}^{2}$$ (10)

Eq (10) is the calculationof the European distance loss function. By calculating the value of $L_{i}^{box}$, the face recognition problem can be converted into a regression problem to simplify the calculation. ${y^{\prime }}_{i}^{\det}$ represents the border coordinates predicted through the network. $y_{i}^{\det}$ represents the actual border coordinates.


$$L_{i}^{mark}={\left\|{y^{\prime }}_{i}^{mark}-y_{i}^{mark}\right\|}_{2}^{2}$$ (11)

**Fig 5:**
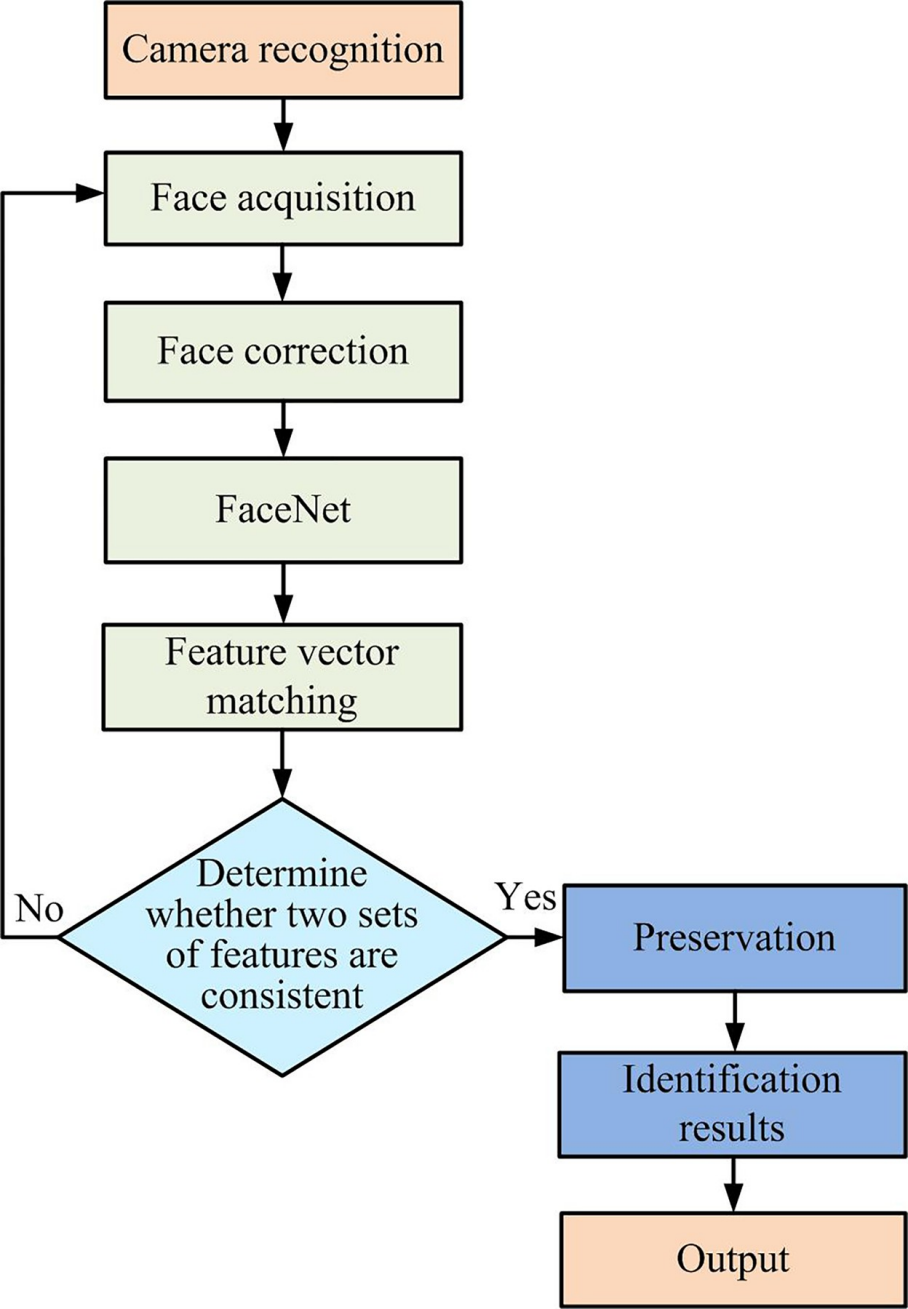
Facial recognition flowchart in FaceNet network.

Eq (11) shows the calculation equation for feature point localization. Eqs (11) and (10) have the same calculation idea, and both adopt the European distance loss function to calculate [24]. ${y^{\prime }}_{i}^{mark}$ and $y_{i}^{mark}$ represent the predicted and actual feature point positions, respectively. After using the MTCNN algorithm to complete facial detection, facial recognition is completed through the FaceNet structure. The facial recognition flowchart under this network structure is listed in Fig 5.

Fig 5 illustrates the facial recognition process within the FaceNet network. Initially, facial images are captured via a camera. Noise may exist within the collected face images due to objective factors such as the brightness of the face collection light and the collection error of the collection device. The collected face images are preprocessed to mitigate such noise. Then, the FaceNet architecture is utilized to extract image features and generate feature vectors, facilitating feature vector matching. Subsequently, a predetermined threshold is established and the image features of the identification image are compared to those of the reference image. If the two features coincide, the image feature information is retained and recognized. Conversely, if the features do not match, a new facial image must be acquired and face recognition performed.

Traditional FaceNet networks encounter problems with large parameter quantities, complex calculations, and high model memory usage, hindering more accurate face recognition. To overcome these issues, the study employs MobileNet as the primary feature network, incorporates the Attention Module and Receptive field block (RFB) module, and utilizes the Mish activation function to optimize the single FaceNet network. The final FaceNet-MobileNet-Fish-Attention-Receptive Field Block(FaceNet-MMAR) model was constructed [25]. Eq (12) is the calculation equation of Mish activation function [26].


$$Mish=x*\tanh(\ln(1+e^{x}))$$ (12)

In Eq (12), tanh represents a hyperbolic tangent function with an output range of -1 to 1. *x* represents a parameter.

**Fig 6:**
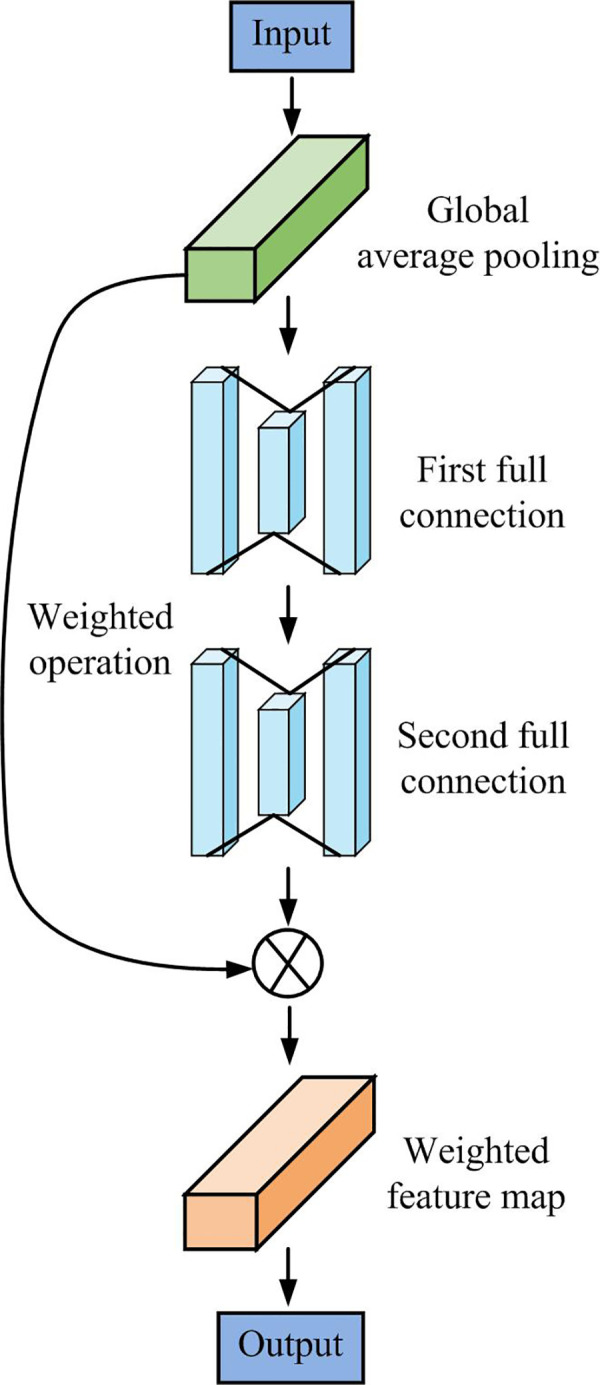
Attention module.

This paper proposes the addition of an attention module based on the main feature network to enhance the efficiency of the backbone network in extracting image features. The input feature layers are weighted, and the network’s feature extraction ability is improved by altering the weight value of each channel [27, 28]. The final layer of the main feature network structure is also augmented with the RFB. Fig 6 illustrates the attention module structure.

**Fig 7:**
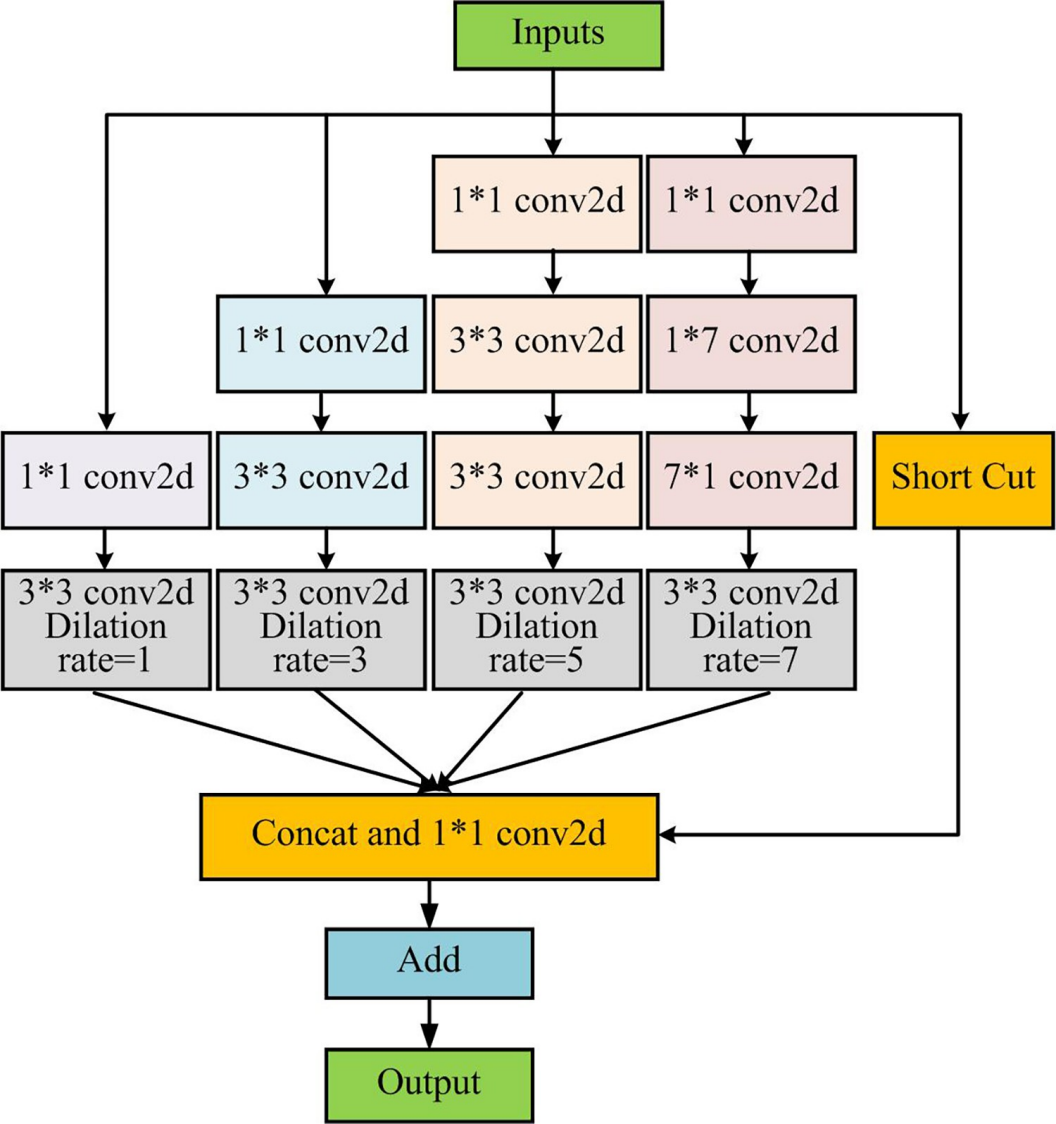
RFB structure.

Fig 6 shows the basic structure of the attention module. In the attention module, the input image features are initially averaged and pooled. This is then followed by two consecutive fully connected operations to ensure that the neuron count in the fully-connected layer matches that of the input feature layer. After completing the connection operation, it is important to apply the sigmoid function to constrain the output value between [0,1]. Then, the final weighted feature map is obtained by multiplying each feature in the input layer by its weight. In the last layer of the primary feature network, an RFB module is added in addition to the attention module to improve the image receptive field and enhance the network’s feature extraction ability. Fig 7 displays the structure of the RFB module.

Fig 7 is a schematic diagram of the RFB structure. There are four different expansion rates, and the Receptive field is expanded through the expansion convolution operation, thus further improving the feature extraction capacity. In addition to setting different expansion rates, this paperalso changed the convolutional kernel size used for extracting featurein the RFB structure to two specifications: 1×7 and 7×1. Compared to the convolutional kernel of 7×7, it can effectively cut down the parameters and simplify the computational complexity. Applying the constructed FaceNet-MMAR model to the access control system of university libraries can successfully complete facial recognition tasks, thus achieving the goal of intelligent library access management.

## IV. Performance analysis of smart face recognition model in university library based on FaceNet MMAR algorithm

The performance of different facial recognition algorithms is first compared from the aspects of feature matching error, loss curve variation, ROC curve, recognition performance, etc. for testing the algorithm. Then, the above model is used in the university’s FR-sys intelligent library to test the application effect of the network model in actual face recognition. It is compared with some common face recognition models to highlight the advantages of this model in practical applications. The results demonstrate that the facial recognition algorithm deployed in this investigation displays excellent performance and applicability.

### A. Performance analysis of FaceNet MMAR algorithm

The essential hardware components are a camera and a computer to establish the experimental environment. A superior quality camera captures the facial images of library users, with its resolution and quality vital for the accuracy and performance of face recognition. Additionally, a high-powered computer must be configured with sufficient computational power and storage capacity to enable the training and implementation of the FaceNet-MMAR algorithm. The specific experimental test environment is shown in Table 1.

**Table 1:** Test environment for research.

Name	Specific model
Video card	GTX 1080ti
Memory	64GB
Processor	Inter Core i7-7700 CPU 3.60GHz
Operating system	Windows 10
Programming language	Java
Data processing platform	Kettle
Visualization Platform	Superset

The hardware equipment for the experiment is given in Table 1. In addition to the hardware equipment, the main software used for this experiment is the TensorFlow framework, the Python programming language, the OpenCV image processor, and the training dataset. The paperwas tested using a homemade face image dataset and a public face image dataset CASIA-WebFace. The two datasets contain samples from different populations, including face images of different ages, genders, races, and appearance characteristics. In addition, both datasets cover face images in different poses, expressions, and lighting conditions, and provide labeling or identity information for each face image to be used for supervised learning during training.To test the effectiveness of FaceNet-MMAR in the intelligent facial recognition model of university libraries, the CASIA-WebFace dataset was used as the experimental dataset for this study to verify the performance of FaceNet-MMAR and FaceNet-MobileNet (FaceNet-MN).

**Fig 8:**
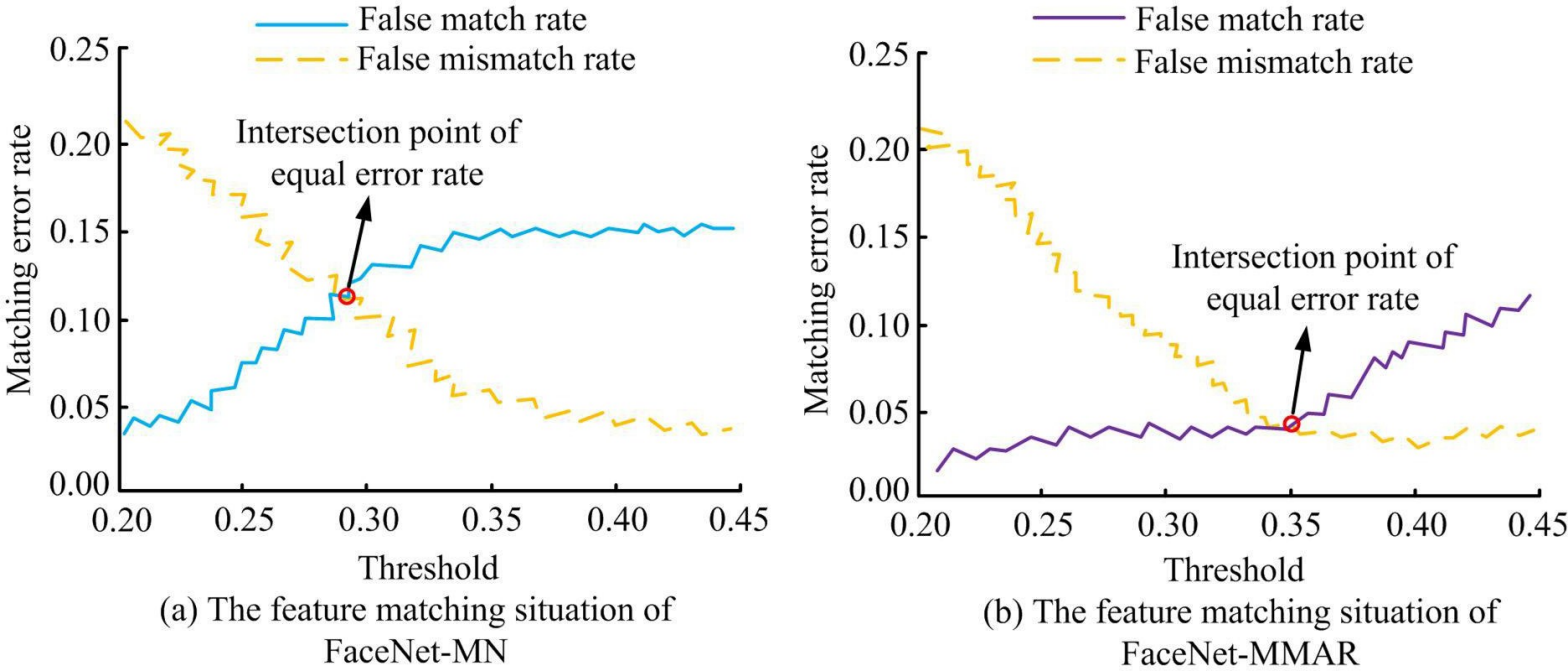
Feature matching errors of different models.

Fig 8 shows the feature matching errors of different models. 8(a) is the feature matching error situation of FaceNet-MN.As the network threshold increases, the matching error rate of the FaceNet-MN network increases, while the non-matching error rate decreases.At a threshold of 0.29, the matching and non-matching error rates intersect, with the matching error value of FaceNet-MN at 0.12. 8(b) shows the feature matching error of FaceNet-MMAR. As the network threshold increases, the error match rate of FaceNet-MMAR continues to increase, but its downward trend is relatively slow. In addition, the error mismatch rate of FaceNet-MMAR also shows a continuous decreasing trend. When the threshold value is 0.35, the error matching rate intersects with the error unmatching rate, and the error matching error value of FaceNet-MMAR is 0.04. Comparing the feature matching error values of the two networks, it was found that the feature matching error value of FaceNet-MMAR in a stable state is much smaller than that of FaceNet-MN. Therefore, FaceNet-MMAR has a better feature matching performance.

**Fig 9:**
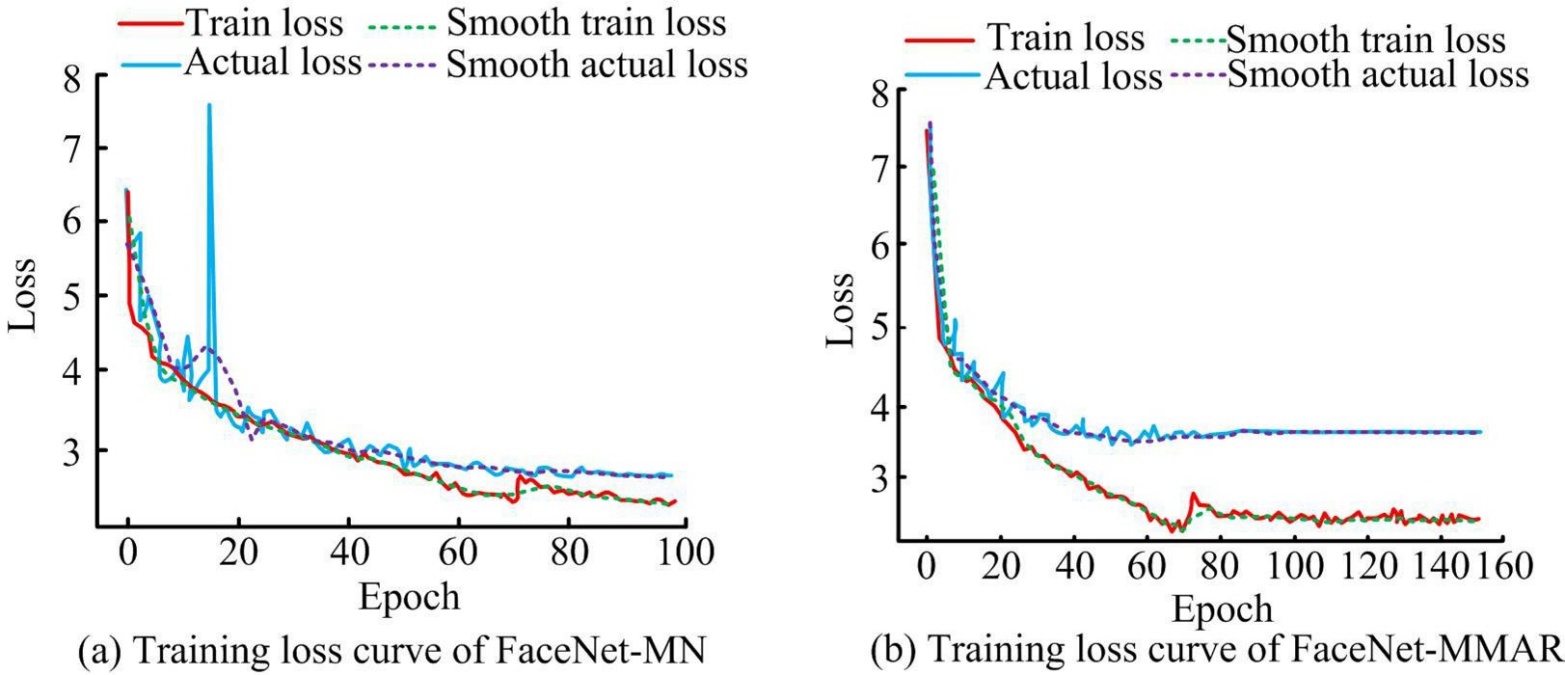
Changes in loss curves of different models.

Fig 9 shows the variation of the loss curves for different models. 9(a) shows the evolution of the loss curve of FaceNet-MN. As theiterations lifts, both the training loss and actual loss curves of FaceNet-MN show a continuous decreasing trend. When the iterations is between 15 and 20, the actual loss curve of the network fluctuates over a large range. 9(b) shows the change in the loss curve of FaceNet-MMAR. As the number of iterations increases, both the training loss and actual loss curves of FaceNet-MMAR exhibit a consistent decline. However, compared to FaceNet-MN, the decline in the loss curve is comparatively smaller. Additionally, the actual loss curve and training loss curve of FaceNet-MMAR display no significant fluctuations during the iteration process. By comparing the two phases, it can be concluded that the FaceNet-MMAR network exhibits more favorable loss performance, implying a greater and more stable influence from iterations on the model.

**Fig 10:**
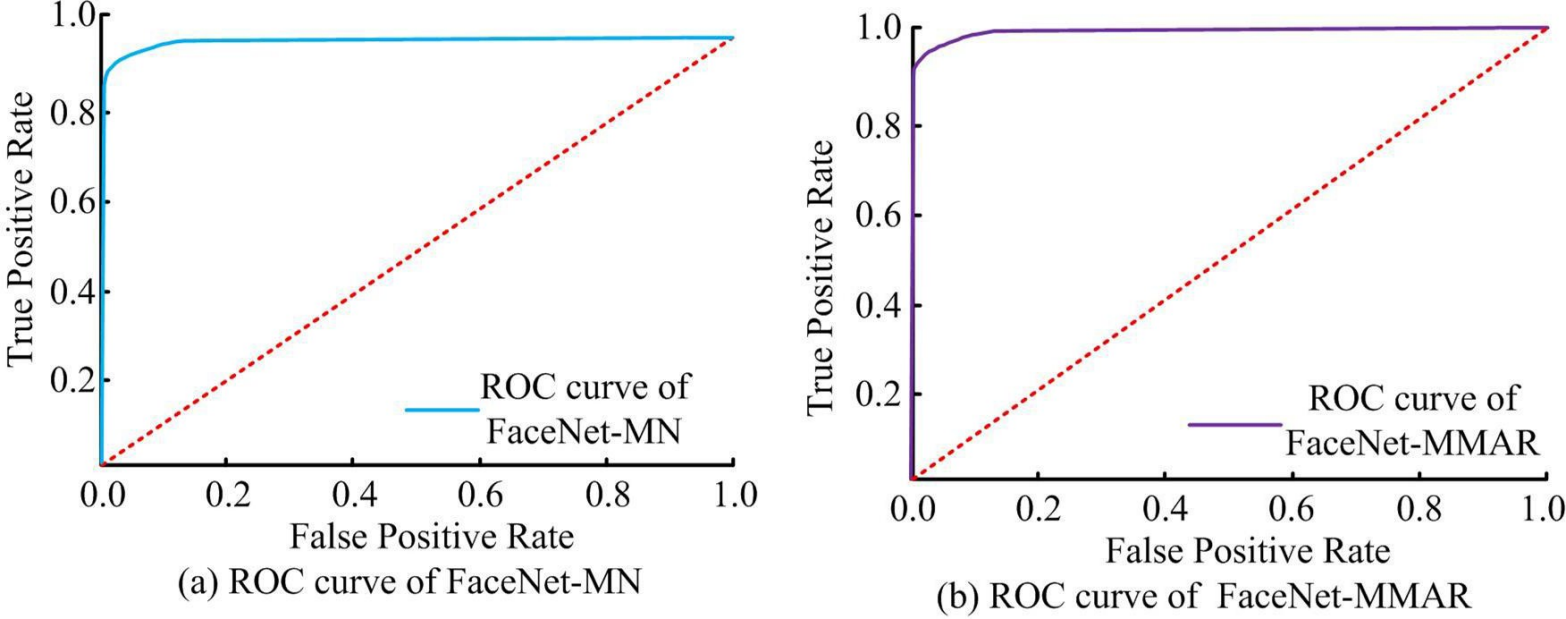
ROC curves of different models.

Fig 10 shows the ROC curves of discriminative models, with (a) the ROC curves of FaceNet-MN and (b) the ROC curves of FaceNet-MMAR. Comparison of the two: FaceNet-MMAR can finally reach a true positive value of 1.0 faster, indicating that its AUC area is larger than that of FaceNet-MN, which can have better recognition accuracy (Shown in Table 2).

**Table 2:** Comparison of recognition performance of different models.

Model	Experimental dataset	Image size of input	Accuracy	Identification error
FaceNet-MN	CASIA-WebFace	160*160	98.56%	1.21%
FaceNet—Attention	CASIA-WebFace	160*160	98.81%	1.06%
FaceNet—Receptive Field Block	CASIA-WebFace	160*160	98.25%	1.58%
FaceNet—Mish	CASIA-WebFace	160*160	98.37%	1.33%
FaceNet-MMAR	CASIA-WebFace	160*160	99.05%	0.51%

Table 1 compares the recognition performance of different models optimized on the basis of FaceNet. The face recognition accuracy of five models, FaceNet-MN, FaceNet Attention, FaceNet RFB, FaceNet-Mish, and FaceNet-MMAR, was compared. The experimental dataset is unified as CASIA-WebFace, with an input image size of 160 * 160. The recognition accuracies of the five face recognition models are 98.56%, 98.81%, 98.25%, 98.37%, and 99.05%, respectively, with recognition errors of 1.21%, 1.06%, 1.58%, 1.33%, and 0.51%. In summary, the FaceNet-MMAR model has the best face recognition accuracy based on FaceNet.

### B. Analysis of the application effect of face recognition model in smart libraries of universities

This chapter is to apply the above model to the university smart library FR-sys, to test the application effect of this network model in practice, and to compare it with some common models to highlight the advantages of this model in practice. The facial data from a smart library at a particular university served as the experimental dataset. 80% of the sample data was used for training, while the remaining 20% served as the validation set.

**Fig 11:**
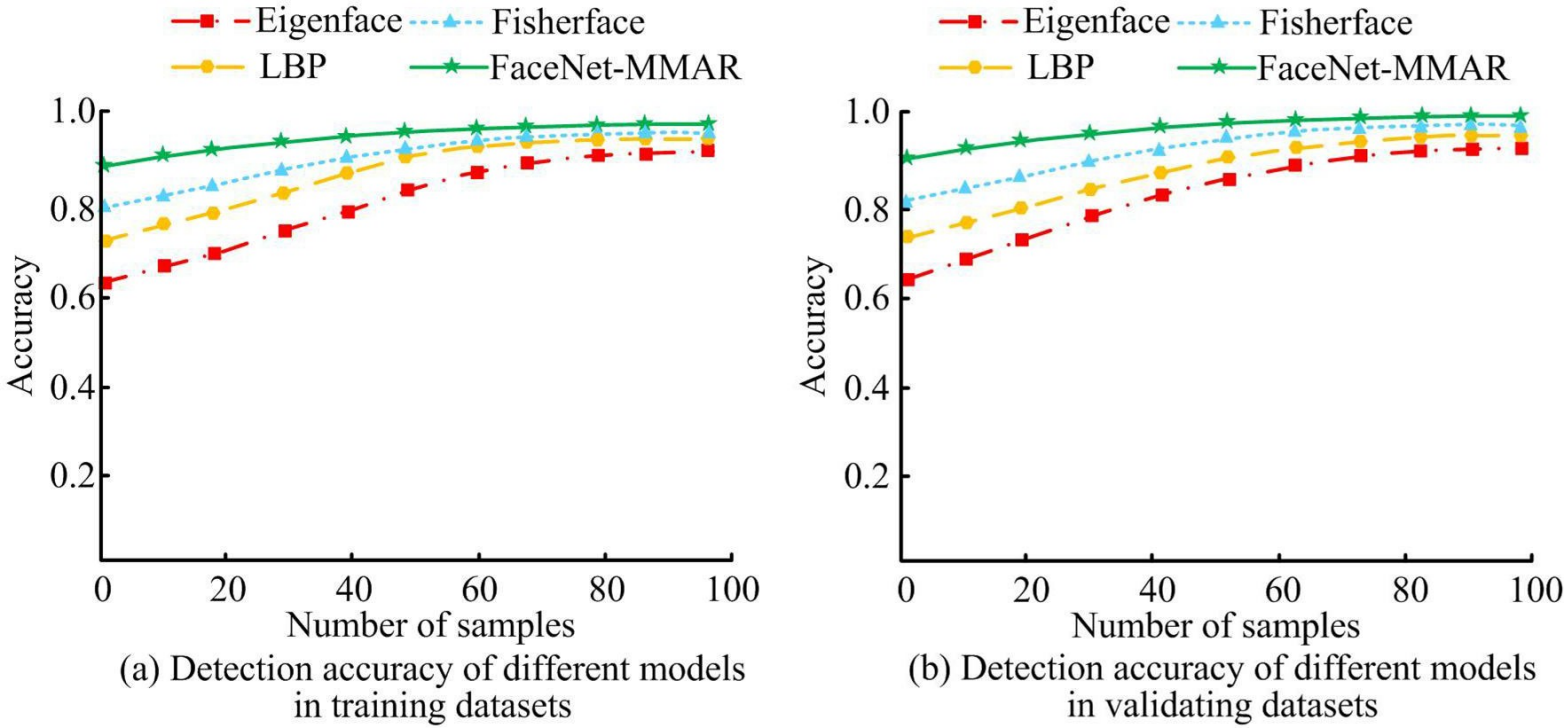
Face recognition accuracy of distinctive ways.

Fig 11 displays the recognition accuracy of different facial recognition methods. In the training dataset of (a), as the sample data continues to increase, the facial recognition accuracy of Eigenface, Local Binary Pattern (LBP), Fisherface, and FaceNet MMAR algorithms shows an upward trend. Among them, the range of change in FaceNet-MMAR is relatively small, while the range of change in Eigenface is relatively large. When the sample size reaches 100, the recognition accuracy of Eigenface, LBP, Fisherface, and FaceNet-MMAR algorithms in the training dataset is 89.56%, 92.34%, 96.57%, and 98.03%, respectively. The validation dataset in Fig 11(B) shows an increasing trend in face recognition accuracy for the four algorithms as the sample size increases. When the sample size is 100, the recognition accuracy of Eigenface, LBP, Fisherface, and FaceNet-MMAR algorithms in the validation dataset is 90.10%, 92.98%, 97.21%, and 99.01%, respectively.

**Fig 12:**
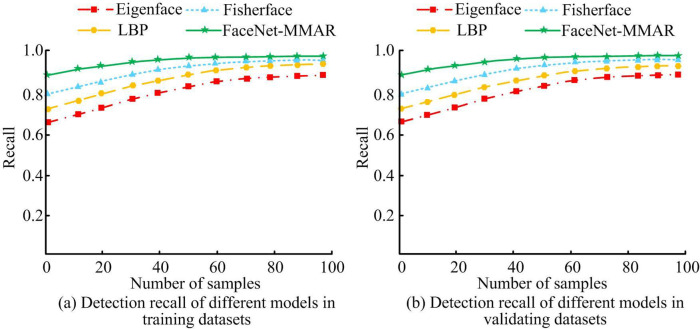
Face recognition recall rates for different methods.

Fig 12 shows the recognition recall rates of different facial recognition methods. From Fig 12(A), in the training dataset, as the sample data continues to increase, the facial recognition recall values of Eigenface, LBP, Fisherface, and FaceNet-MMAR all show a continuous upward trend. When the sample size is 100, the recall rates of Eigenface, LBP, Fisherface, and FaceNet-MMAR in the training dataset are 88.26%, 91.76%, 96.23%, and 98.01%, respectively. In the validation dataset of Fig 12(B), as the sample data continues to increase, the recall values of the four algorithms are still increasing. When the sample size is 100, the recall rates of Eigenface, LBP, Fisherface, and FaceNet-MMAR in the validation dataset are 89.21%, 90.65%, 96.59%, and 98.98%, respectively.

**Fig 13:**
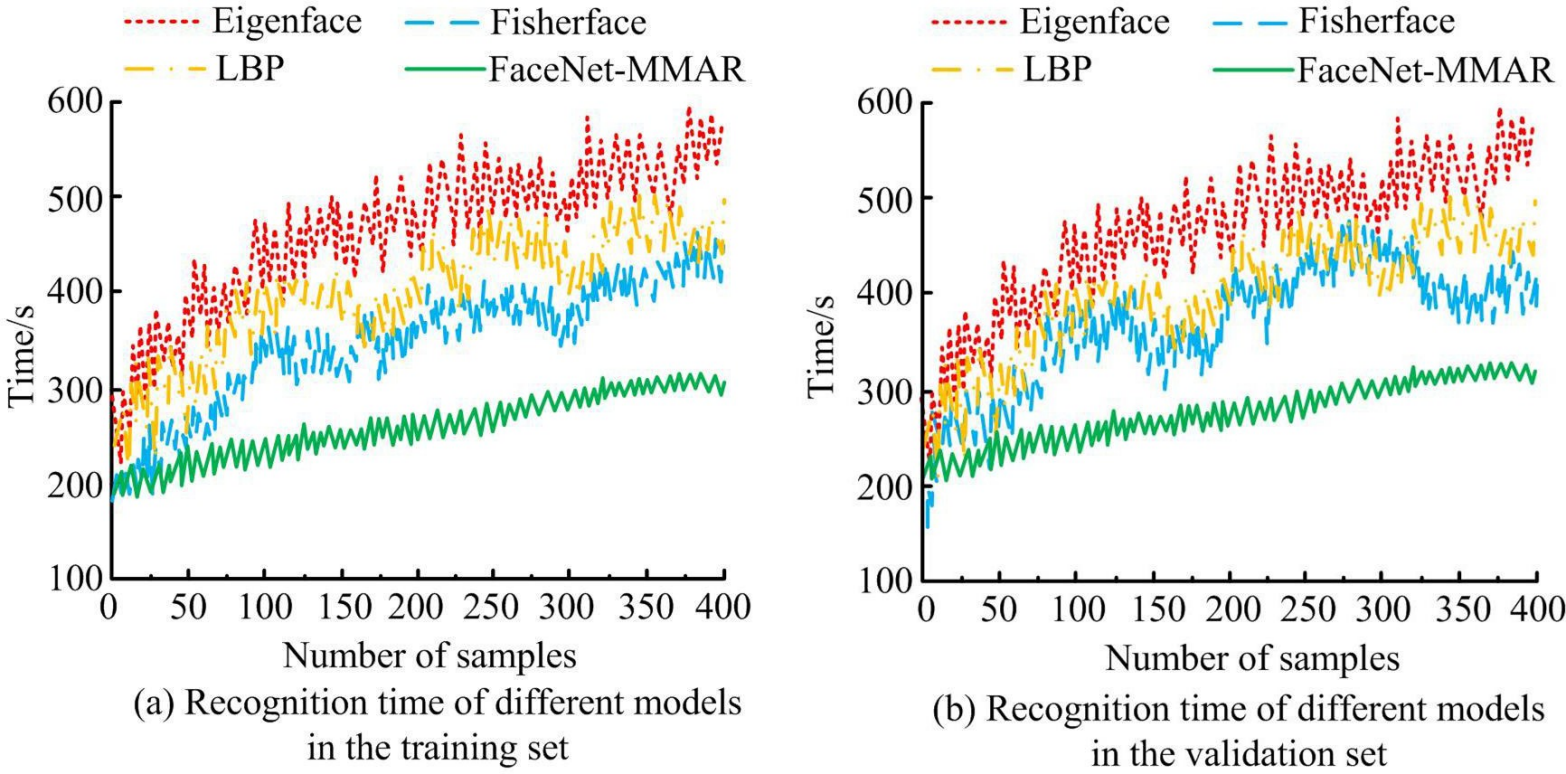
Time spent on recognizing faces using different methods.

Fig 13 shows the time taken to recognize faces using different methods. In the training dataset of 13 (a), the recognition time of all four face recognition algorithms increases as the sample size increases. As the sample size increased from 0 to 400, the time spent by Eigenface, LBP, Fisherface, and FaceNet-MMAR increased from 286s, 261s, 210s, and 196s to 586s, 465s, 413s, and 284s, respectively. In addition, Eigenface, LBP, and Fisherface experience significant fluctuations in recognition time, while FaceNet-MMAR is within a relatively stable range. Similarly, the performance of the four algorithms in the validation set is displayed in Fig 13(B). In summary, FaceNet-MMAR not only reduces recognition time but also demonstrates a consistently stable fluctuation in facial recognition time. This suggests that FaceNet-MMAR outperforms the other three methods in terms of recognition efficiency.

**Fig 14:**
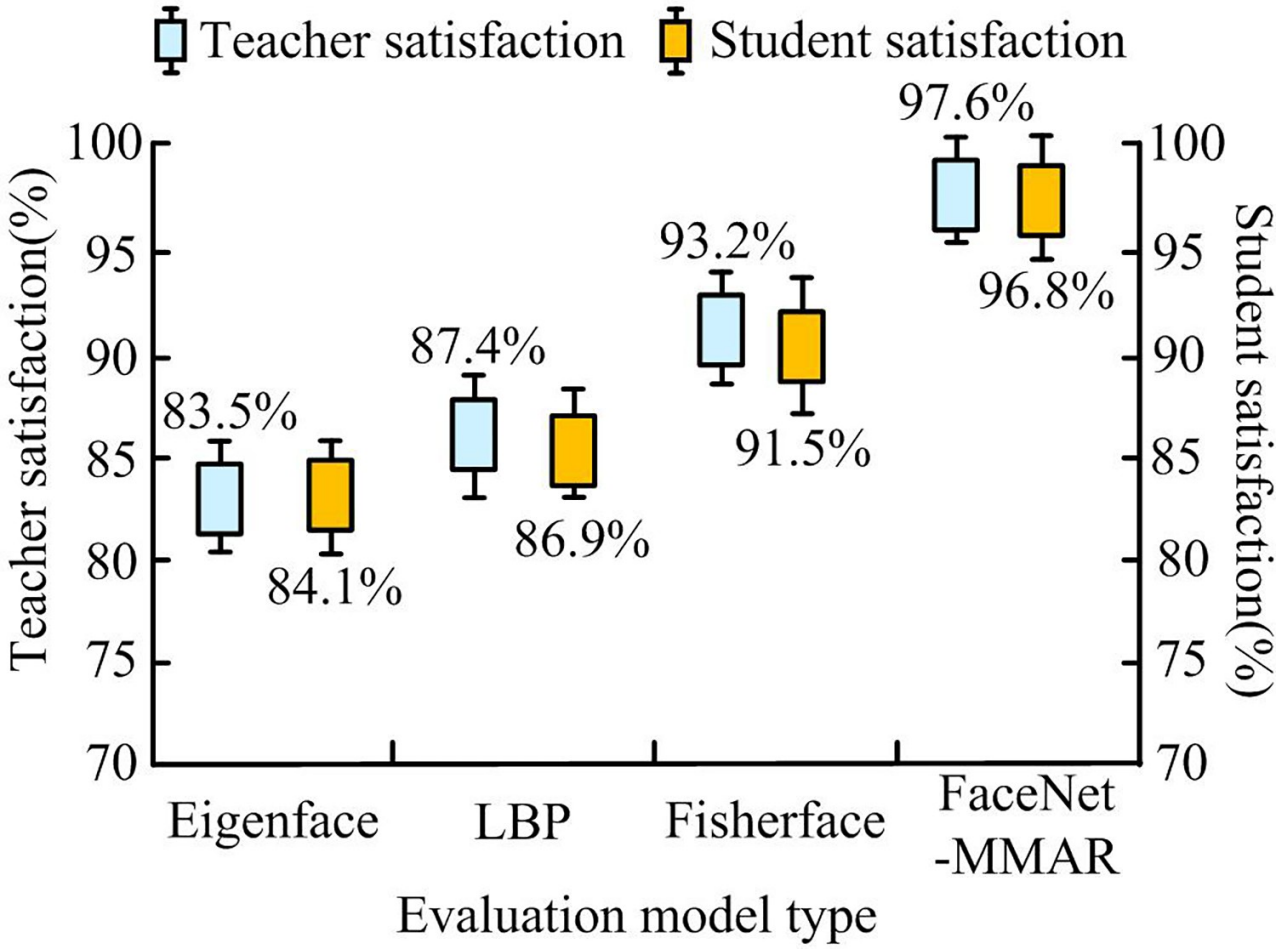
Satisfaction of university teachers and students with various face recognition algorithms.

Fig 14 shows the satisfaction statistics of university teachers and students with various facial recognition algorithms. For traditional Eigenface and LBP, the satisfaction rates for teachers and students were 83.5% and 84.1%, and 87.4% and 86.9%, respectively. For Fisherface, the satisfaction rates were 93.2% and 91.5%, respectively. The FaceNet-MMAR algorithm achieved 97.6% teacher satisfaction and 96.8% student satisfaction. This method not only outperforms other comparative methods in performance, but also can satisfy more teachers and students.

**Fig 15:**
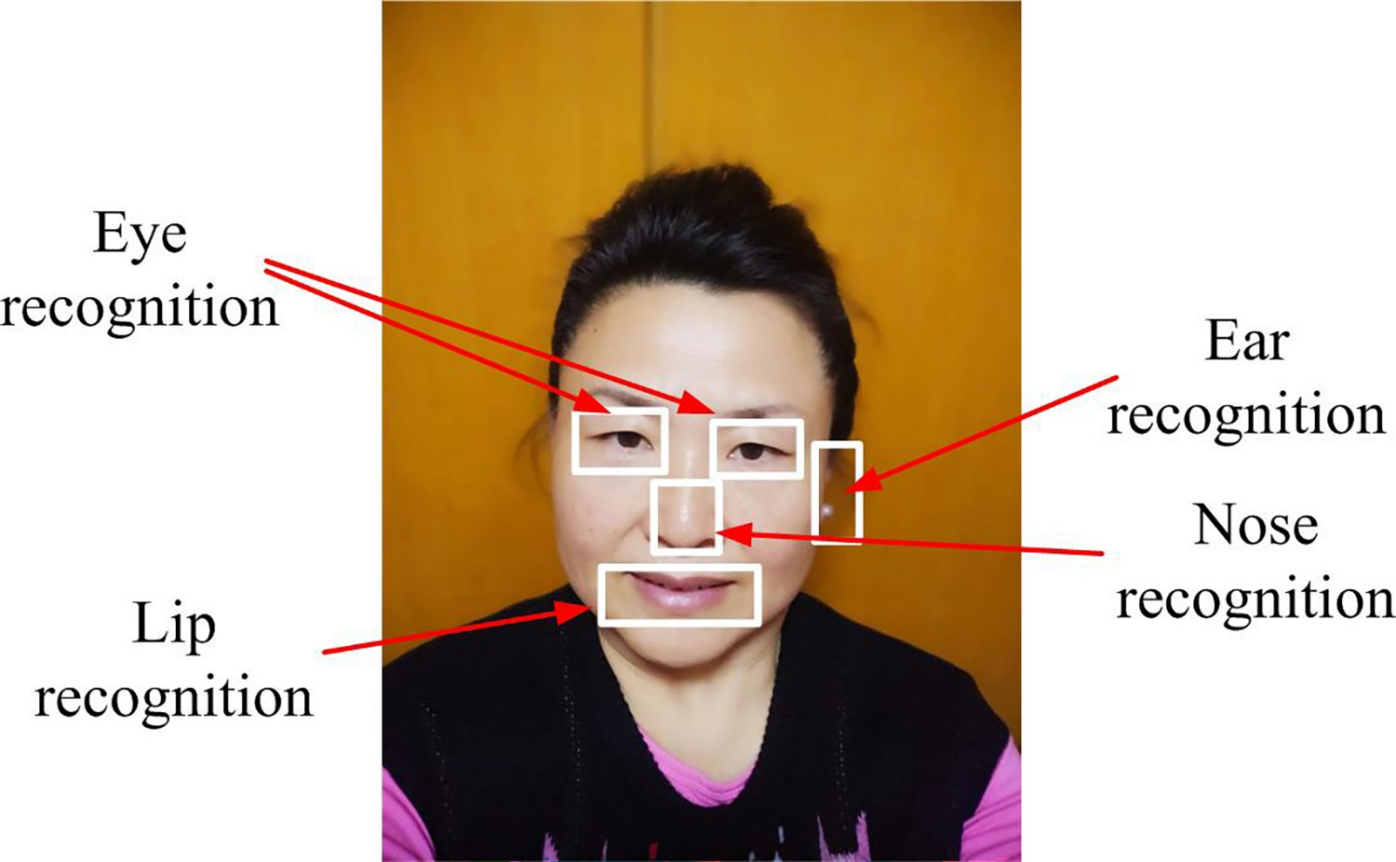
Visualization results of face recognition.

Fig 15 shows the visualization analysis results of facial recognition of library faces using FaceNet-MMAR face recognition model. The model can successfully identify the five distinct facial features of different areas, resulting in a more effective access control recognition. Applying the above face recognition model to the face recognition system of the intelligent library at the university has a greater effect and can ensure greater accuracy and effectiveness for library access control recognition.

## V. Conclusion

This study is based on the FaceNet network and optimized by adding attention mechanisms, RFB, and other neural network structures to strengthen the recognition accuracy and traditional facial recognition efficiency. The results verified that compared with FaceNet-MN, the error matching error value of FaceNet-MMAR is 0.04, which is much smaller than FaceNet-MN’s 0.12. When the model is iterated between 15 and 20 times, FaceNet-MN exhibits significant fluctuations, while the loss curve of FaceNet-MMAR is relatively flat. On the same dataset, the facial recognition accuracy of FaceNet-MN, FaceNet-Attention, FaceNet-RFB, FaceNet-Mish, and FaceNet-MMAR were 98.56%, 98.81%, 98.25%, 98.37%, and 99.05%, respectively, with recognition errors of 1.21%, 1.06%, 1.58%, 1.33%, and 0.51%. This indicates that on the basis of FaceNet, the FaceNet-MMAR model has the best face recognition accuracy.The application of this model to the management system of smart libraries in universities found that FaceNet-MMAR performs better in facial recognition accuracy and recall than the other three traditional algorithms in both the training and validation sets. In addition, when the sample size is 400, the recognition time of FaceNet-MMAR is only 284 seconds, which is much lower than other algorithms. In the end, FaceNet-MMAR achieved 97.6% teacher satisfaction and 96.8% student satisfaction, respectively. Although the FaceNet-MMAR constructed by this paperhas good performance and practical application results, there are still some errors in the testing process due to the insufficient number of data samples and the insufficient richness of sample features. Further research can consider using more experimental datasets to detect themodel performance.
